# Engaging Nurses in Research for a Randomized Clinical Trial of a Behavioral Health Intervention

**DOI:** 10.1155/2013/183984

**Published:** 2013-09-11

**Authors:** Lona Roll, Kristin Stegenga, Verna Hendricks-Ferguson, Yvonne J. Barnes, Brooke Cherven, Sharron L. Docherty, Sheri L. Robb, Joan E. Haase

**Affiliations:** ^1^The University of Texas Health Science Center at San Antonio, 333 North Santa Rosa, San Antonio, TX 78207, USA; ^2^Children's Mercy Hospital, 2401 Gillham Road, Kansas City, MO 64108, USA; ^3^St. Louis University School of Nursing, 3525 Caroline Street, St. Louis, MO 63104-1099, USA; ^4^Washington University School of Medicine, One Children's Place, St. Louis, MO 63110, USA; ^5^Children's Healthcare of Atlanta, 2015 Uppergate Drive, Atlanta, GA 30322, USA; ^6^Duke University School of Nursing, DUMC 3322, 307 Trent Drive, Durham, NC 27710, USA; ^7^Indiana University School of Nursing, 1111 Middle Drive NU 439W, Indianapolis, IN 46202-5107, USA

## Abstract

Nurse involvement in research is essential to the expansion of nursing science and improved care for patients. The research participation challenges encountered by nurses providing direct care (direct care nurses) include balancing patient care demands with research, adjusting to fluctuating staff and patient volumes, working with interdisciplinary personnel, and feeling comfortable with their knowledge of the research process. The purpose of this paper is to describe efforts to engage nurses in research for the Stories and Music for Adolescent/Young Adult Resilience during Transplant (SMART) study. SMART was an NIH-funded, multisite, randomized, behavioral clinical trial of a music therapy intervention for adolescents/young adults (AYA) undergoing stem cell transplant for an oncology condition. The study was conducted at 8 sites by a large multidisciplinary team that included direct care nurses, advanced practice nurses, and nurse researchers, as well as board-certified music therapists, clinical research coordinators, and physicians. Efforts to include direct care nurses in the conduct of this study fostered mutual respect across disciplines in both academic and clinical settings.

## 1. Introduction

Over the years, there have been many recommendations to close the research/practice gap, including efforts to increase the availability and applicability of the evidence for practice through the involvement of direct care nurses on research teams [1–5]. Academic nurse researchers have had an important role in advancing evidence-based practice; unfortunately, direct care nurses are frequently absent from the research team [6]. The American Nurses Association (ANA) advocates nurse involvement in research, stating, “all nurses working alone or in collaboration with others can participate in the advancement of the profession through the development, evaluation, dissemination, and application of knowledge in practice” [7]. 

Having opportunities to observe and engage in ongoing research is the key to encourage nurses' participation in research [6]. Yet, full involvement of direct care nurses in the conduct of clinical research is challenging because these nurses often lack the knowledge/skills, support, and time needed for broad immersion [6, 8]. A deeper understanding of some of these challenges, as well as possible solutions, may assist in fostering staff nurses' involvement in clinical research. This paper reports on observations of direct care nurse engagement in a large multisite, behavioral intervention study and strategies employed to foster and maintain that engagement.

## 2. Background

 Adolescents and young adults (AYA) undergoing hematopoetic stem cell transplant (HSCT) for treatment of cancer have a long, difficult journey. The typical transplant course involves (a) a pretransplant conditioning regimen with chemotherapy +/− radiation lasting 2–10 days; (b) the stem-cell infusion; and (c) posttransplant recovery lasting days to months. Side effects during the transplant course can be severe and include nausea/vomiting, diarrhea, mouth sores, infection, kidney/bladder problems, lung problems, fatigue, pain, and graft versus host disease. AYA experiencing this process are challenged to endure this complex treatment and find a balance between enduring the transplant and side effects and continuing the normal process development. The Stories and Music for Adolescent/Young Adult Resilience during Transplant (SMART) study was developed and implemented with the goal of identifying a way to assist AYA through the transplant process with a positive psychosocial outcome. 

The SMART study was a randomized Children's Oncology Group clinical trial (ANUR0631) supported by NIH-NINR and NCI designed to evaluate the efficacy of a therapeutic music (TMV) intervention compared to a low-dose audio book control intervention for AYA 11–24 years of age undergoing HSCT for an oncology condition. Complexities implementing this study included the following: (a) a large disciplinary team required for implementation; (b) extensive outcome measures; (c) multisession TMV intervention during the transplant course to AYA experiencing significant physical symptoms; and (d) an extensive quality assurance program to ensure consistent and accurate implementation of the study. 

 As the SMART study was implemented, the research team held conference calls twice a month to discuss study implementation and to solve problem barriers. We recognized the need to keep all members on each HSCT unit informed and engaged with study activities. The team observed that the complex nature of the HSCT combined with the intense process of study implementation required the cooperation and participation of HSCT direct care nurses. Through our discussions, we identified key areas in which we needed the nurses collaboration. These included (a) informing study staff of patient clinical status; (b) organizing nursing care and symptom management to maximize ability of study participation to complete study activities; (c) supporting and encouraging patient participation; and (d) following quality assurance procedures to maintain evaluator blinding during the intervention. The purpose of this paper is to describe the efforts of the core research team to engage and include direct care nurses at each study site in the conduct of the study. 

## 3. Challenges

Multiple challenges related to the successful inclusion of direct care nurses into the research process were encountered during study implementation. The main challenges our research team encountered were consistent with those identified in the literature [6, 8]. The first broad challenge related to *time*, which included balancing patient care demands with research needs and adjusting to fluctuating staff and patient volumes. The second key challenge was *support*, which included coordinating clinical and study personnel. Third, *skills/knowledge* included ensuring that direct care nurses had appropriate levels of knowledge and comfort with behavioral intervention research. We describe each of these challenges in detail below. 

### 3.1. Time

#### 3.1.1. Balancing Patient Care Demands with Research

The targeted population, AYA undergoing an SCT, had high acuity and experienced multiple distressing physical symptoms including pain, mouth sores, nausea and vomiting, diarrhea, and fatigue. Sometimes, limited time and complex patient care needs made it difficult for nurses to accomplish necessary direct care while also supporting the research process. This experience is consistent with literature indicating that nurses' primary concern is delivery of timely, competent care and being an advocate for patients; hence, anything perceived as not in the patients' best interest, such as involvement in research, will likely be abandoned [9]. To remain involved, the direct care nurses needed guidance in how to find ways to balance provision of quality patient care along with adherence to correct delivery of the study protocol. In order to address this concern and sustain involvement of the direct care nurses, our research team realized that we would need to develop and implement ongoing booster training to introduce strategies to assist the nurses in how to adhere to the research protocol while also remaining sensitive and responsive to the ever-changing needs of the high acuity patient population. 

#### 3.1.2. Adjusting to Fluctuating Staff and Patient Volumes

Along with high patient acuity, fluctuations in nurse staffing patterns created an additional challenge for nurse involvement in the research process. Staffing patterns in clinical settings require that direct care nurses may be unpredictability assigned on a daily basis to work in different in-patient care units to accommodate changes in daily patient census patterns and acuity. For instance, nurses familiar with the study, who knew how to integrate study activities into patients' care plans, were often reassigned to work in other in-patient care units when the HCST patient census was lower. Conversely, in times of high HCST patient census patterns, direct care nurses that were not familiar with the research study may be reassigned to provide patient care to AYA in the HSCT units where the study was being conducted. The number of study-eligible patients would also fluctuate; for example, there were times when study approved clinical sites had multiple patients simultaneously enrolled in the study and other time periods in which no eligible patients could be recruited to the study. After periods of prolonged study inactivity, direct care nurses often felt less prepared to support the research protocol because they lacked recent experience integrating study procedures into routine clinical care. For example, obtaining physiological data regarding aspects of the patients' status (e.g., pain and mucositis) to provide to the study interveners and coordinating patient care (e.g., timing of pain medication delivery in relation to scheduled intervention sessions) was less intuitive after periods of study inactivity. It became evident that fluctuations in nursing staff and study participant volumes were affecting the direct care nurses' enthusiasm and focused commitment to be actively engaged in recommended recruitment activities (e.g., offering a study brochure to eligible patients) and that the nurses would require a booster training plan for ongoing support and education. 

### 3.2. Support

*Coordinating Interdisciplinary Clinical and Study Personnel.* Coordination of clinical and study personnel was a primary challenge encountered during SMART study implementation. Study personnel included HSCT coordinators who identified study-eligible patients, project managers who obtained informed consent and scheduled study activities, clinical research coordinators who administered study measures, and board-certified music therapists who delivered the study intervention sessions. Some study team members were not familiar with HSCT patients' usual clinical pattern and the nature of their changing needs. Therefore, some of our study team members often needed to consult with the direct care nurses about the current health care status of enrolled patients and explore ways to integrate intervention sessions and measurement times into the daily flow of the patients' plans of care. Consistent and effective communication among the direct care nurses and study team members fostered successful scheduling and execution of the study sessions and activities during delivery of the enrolled patients' HSCT treatments. 

### 3.3. Skills/Knowledge

*Ensuring Appropriate Levels of Knowledge and Comfort with Behavioral Intervention Research.* Behavioral intervention studies are not commonly conducted in pediatric HSCT clinical settings. However, pediatric nurses practicing in Children's Oncology Group HSCT units often have experience in assisting physicians with the conduct of RCT studies designed to evaluate the efficacy of specific medications and treatment regimens. The role of direct care nurses in such RCT studies often involves familiar skills such as drawing blood for laboratory specimens, administering medications in a specified manner, and scheduling prescribed radiologic evaluations. Generally, direct care nurses have little or no exposure to nurse researchers who are conducting behavioral intervention studies. Thus, direct care nurses needed additional information to understand how behavioral intervention studies differ from clinical trials in terms of study design, procedures for ensuring intervention and evaluation integrity, data collection methods, and expected outcomes. 

In the SMART study, our team observed that when direct care nurses did not understand behavioral intervention study features, the nurses were uncertain about how to support intervention and data collection processes. For example, the study evaluators needed to be blinded (or uninformed) to which intervention arm that the enrolled participants were randomized to receive. Because the evaluators were not inadvertently unblinded, the direct care nurses had to be careful about what was discussed with evaluators while also being asked to provide evaluators with specific data about participants' symptom experiences prior to the AYA's intervention sessions. Such support and help by direct care nurses were critical to the integrity of the study. 

Identifying the study-related challenges that direct care nurses might experience was one key factor to successfully initiating and sustaining these nurses' ongoing commitment and engagement in the study. The second key factor, described below, was developing strategies to address and overcome the identified challenges.

## 4. Strategies

 Throughout the study, SMART study personnel worked to address the challenges of conducting interdisciplinary, multisite behavioral intervention research within HSCT units. Some of the previously identified challenges could be anticipated and planned for, whereas others were unexpected and required quick and timely communication among the research team members. In this section, we outline some of the “a priori” strategies that were used for anticipated protocol challenges, as well as strategies developed in response to unexpected study challenges. Each strategy addressed the key challenges identified above. Training and ongoing research team discussions were targeted at (a) reducing protocol implementation challenges by improving study-related knowledge and skills, (b) preventing time-related challenges by integrating study procedures into the AYA's plans of care, and (c) improving study protocol adherence by generating and maintaining enthusiasm for the study. It is important to note that while each of these strategies was aimed at preventing or dealing with a specific study-related challenge, addressing one challenge inevitably addressed others. For instance, provision of education about the study protocol and ongoing relevant team communication also supported nurses' commitment to consistent protocol delivery, and strategies to support integration of study procedures into enrolled patients' plans of care also enabled the patients' assigned nurses to more easily integrate study activities into patient care. 

### 4.1. Training

To ensure success during the start-up phase of the SMART study, a first priority was to ensure that study personnel understood and could implement the study appropriately. Prior to initiating the SMART trial, all study personnel attended team-building and educational meetings that provided extensive training on the study design and protocols, the SCT process, and the roles of all team members. These two-day meetings provided necessary foundational information and support prior to bringing the study into the clinical settings. Written manuals for each team member were developed to provide consistency and reinforce content taught during training. In addition, at each site nurses in advanced practice roles were identified and received salary support and training to assist with study coordination and integration into the clinical setting. After training, the study personnel were responsible for presenting study information to the nursing staff at their respective clinical sites. Strategies to reach other clinical personnel at each site and maintain study visibility included presentations at regularly scheduled unit-based staff meetings, informal staff meetings held on all shifts, and distribution of study information through in-service fliers, study brochures, and posters.

### 4.2. Regular and Relevant Communication

To keep the direct care nurses informed of the SMART study progress we used several communication strategies. Updates on accrual, attrition, and timeliness of evaluation were provided to all study personnel on a regular basis, and these updates were then communicated to the nursing staff of the participating units. Project managers also communicated with direct care nurses on the HSCT units whenever enrollment of new patients into the study was anticipated. Quarterly study newsletters published to celebrate all team members' achievements included professional accomplishments such as promotions, presentations, and publications, as well as personal celebrations such as weddings and births of children and grandchildren. Each site was invited to submit personal or professional items to be showcased in the newsletters by all study personnel at each data collection site. The inclusion of pictures in the newsletters helped team members and direct care nursing staff connect names and faces of team members across participating sites. These newsletters were compiled at the lead site by the project manager and made available to all nursing staff at each site. As a whole, these strategies promoted continued interest, consistent communication, and pride in all study-related accomplishments. 

Communication was also fostered through the use of regularly scheduled conference calls for the study staff. The conference calls were scheduled biweekly to foster study personnel sharing of perspectives, ideas, challenges, and progress related to study activities across sites. By providing a mechanism for group participation in problem-solving, study staff members were able to use the collective wisdom of the group to prevent or address any impediments to smooth study implementation at their respective sites. The study coordinator at each site also scheduled team meetings with the HSCT personnel to provide timely study related information and suggestions for overcoming any identified obstacles related to study flow at individual sites. The site meetings provided a venue for direct care nurses to have an opportunity to share their perspectives and suggestions about strategies to be considered in order to improve implementation of the study at their respective sites, and the meetings fostered collaborative partnerships and helped to maintain study integrity. 

### 4.3. Integration of Study Procedures into Patient Care

Another important way was that commitment to the study was fostered to integrate study procedures into the patients' daily plans of care. As previously discussed, this was especially important because patients in this study were often quite sick. Academic and study nurses collaborated with the direct care nurses to develop a SMART study nursing care plan as a communication tool for study personnel (see Supplementary Figure 1 available online at http://dx.doi.org/10.1155/2013/183984). This care plan was developed by the project manager and direct care nurses at one study site and shared with all other sites. This care plan showcased specific dates and times for scheduled study interventions and evaluations so that the direct care nurses could anticipate and plan care for their patients while simultaneously facilitating the completion of study activities. This care plan also provided a reference point that assisted the direct care nurses to remember to share key information about the health care status of enrolled patients with the clinical research coordinators. In addition to encouraging integration of study interventions into daily care, the care plan enhanced the direct care nurses' confidence in study facilitation and participation.

### 4.4. Generating and Maintaining Enthusiasm

Enthusiasm for the study was also generated by establishing the legitimacy and importance of the study through presentations at professional conferences attended by nurse clinicians. Of particular importance were presentations and updates about the SMART study that were made at semiannual Children's Oncology Group meetings, which were attended by participating site physicians, nurses, and clinical research associates. These regular updates became an excellent vehicle for communication about the study and spurred interest and enthusiasm from other nonparticipating sites. In addition, sessions at the Association of Pediatric Hematology Oncology Nurses meetings gave nurses across the country an opportunity to learn about the study and its progress. Following these presentations, nurses from participating sites often expressed pride in being a part of the study. 

Posters and symposia on multiple aspects of the study were presented at meetings of other groups, including the International Pediatric Transplant Association, International Society of Pediatric Oncology, International Psychosocial Oncology Society, American Psychosocial Oncology Society, American Music Therapy Association, American Cancer Society/Oncology Nurses Society National Conference on Cancer Nursing, Midwest Nursing Research Society, International Institute for Qualitative Methodologies Conference, and Bone Marrow Transplant Tandem Meetings. The level of enthusiasm of the study team was reflected in their involvement in multiple dissemination efforts, including presentations, published abstracts, and manuscripts [10–22]. These efforts afforded team members opportunities to participate in dissemination, a new and exciting opportunity for many of the participating nurses that was also celebrated at each of the data collection sites. The wide variety of venues made it possible for the nurses involved with the SMART study to understand the significance of their contribution to the development of nursing science. 

The SMART intervention also included specific activities that fostered team sharing and enthusiasm by the nursing staff at each study site. Examples of these activities included (a) AYA participants randomized to the TMV intervention created music videos and often included nursing staff in the content of their videos, (b) the last TMV intervention session included a video premiere viewing party to which nursing staff members were often invited, and (c) during the video premier, nursing staff could view the final product of the TMV intervention and witness the positive effect it had on the AYA. These activities afforded direct care nurses the opportunity to witness the important impact they were making in the lives of their patients through their participation in a behavioral intervention research study.

## 5. Conclusion

Identifying challenges to nurse participation in a behavioral intervention study and developing strategies to address those challenges were critical to successful implementation of the SMART trial. While we did not formally evaluate our processes for including direct care nurses in the research process, a few key points emerged. Successful implementation of a research project requires a commitment to problem-solving and effective communication. The ability to foster these two tenets can lead to mutual respect and cooperation among key study personnel and nursing staff. Engaging stakeholders from both “sides” ahead of implementation allows for troubleshooting. Open dialogue along the way keeps challenging situations from becoming more problematic. A simple, yet key strategy was ongoing and responsive communication. Building rapport with staff on both the transplant unit and individual levels at each site allowed for timely identification of challenges and enabled staff to feel comfortable reaching out when they needed help. Anecdotal interactions with the nursing staff fostered early identification of potential or actual challenges that arose periodically during study implementation; however, being able to observe how AYA during HCST were still able to complete the project and feeling that the study staff were there to support them made the direct care nurses eager to be a part of the team. 

Direct care nurses involved in the SMART study became more familiar with the conduct of behavioral intervention research, observed the benefits of patient and family participation, and had opportunities for career advancement through participation in professional presentations and publications, but perhaps most importantly these nurses made a significant contribution to advancing science to improve patient care. Direct care nurse participation in study implementation is essential to the success of behavioral intervention research, and their full participation will result in research products that are more readily translated into practice. Nurse researchers need to address the inclusion of direct care nurses as new research is planned and developed. Fostering a culture of respect for and accessibility to research is vital to continued efforts to advance evidence-based nursing practice [23, 24].

## Supplementary Material

The SMART Study Care Plan is a communication tool developed by academic and study nurses to provide a roadmap for staff nurses caring for patients enrolled on the SMART study. This care plan assists the nurse in integrating study activities into the daily clinical care of the patient.
