# Supplementary material for: Engaging Nurses in Research for a Randomized Clinical Trial of a Behavioral Health Intervention
Source: Nurs Res Pract. 2013 Sep 11;2013:183984. doi: 10.1155/2013/183984 (PMC3786524; doi:10.1155/2013/183984)
Supplement: Supplementary file 1 — The SMART Study Care Plan is a communication tool developed by academic and study nurses to provide a roadmap for staff nurses caring for patients enrolled on the SMART study. This care plan assists the nurse in integrating study activities into the daily clinical care of the patient. [file 183984.f1.docx]

Figure 1:

**
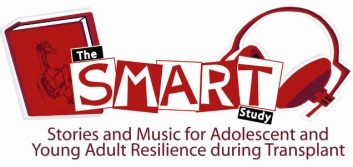
**

**SMART STUDY CARE PLAN**

____________________ is enrolled on the SMART Study ANUR0631 (Stories and Music for Adolescent/Young Adult Resilience during Transplant.

An Intervener (music therapist) and an Evaluator will be spending time with ______________at the following time points:

| INTERVENTIONS |  |  | Approximate Date/Time |
| --- | --- | --- | --- |
| Week 1 | Session 1 | 60 minutes |  |
|  | Session 2 | 60 minutes |  |
| Week 2 | Session 3 | 60 minutes |  |
|  | Session 4 | 60 minutes |  |
| Week 3 | Session 5 | 60 minutes |  |
|  | Session 6 | 60 minutes |  |
| EVALUATIONS |  |  |  |
| Week 1 | Session 2 |  |  |
|  | Pre-intervention | 15 minutes |  |
|  | Post intervention | 15 minutes |  |
| Week 2 | Session 4 |  |  |
|  | Pre-intervention | 15 minutes |  |
|  | Post intervention | 15 minutes |  |
| Week 3 | Session 6 |  |  |
|  | Pre-intervention | 15 minutes |  |
|  | Post intervention | 15 minutes |  |
| Week 3 | Session 6 Time 2 | 60 minutes |  |

Nursing Actions:

- Treat evaluation and intervention time as part of the daily schedule.
- Try to schedule procedures/medications to minimize interruptions and maximize ________________ability to actively participate.
- Be prepared to provide the evaluator with assessment data on mucositis and sedation for pre intervention time points at session 2, 4, 6.
- To preserve the integrity of the study, DO NOT reveal to the evaluator which intervention group _____________is in.
- Encourage the adolescent/family not to discuss this study with other patients or families.
- Nursing interaction with the study participant in the book on tape group should be limited to discussion about the book itself.
- Nursing interaction with the study participant in the music video group can be discussion about what they are doing with their project and how they are feeling about the process. These interactions are encouraged.

If the study participant/family/nurses or other caregivers have any concerns about any aspect of the study, please notify: _____________________________
